# A Process-Centric Survey of AI for Scientific Discovery Through the EXHYTE Framework

**DOI:** 10.21203/rs.3.rs-8370059/v1

**Published:** 2025-12-17

**Authors:** Md Musaddaqul Hasib, Sumin Jo, Harsh Sinha, Jifeng Song, Arun Das, Zhentao Liu, Hugh Galloway, Huey Huang, Kexun Zhang, Shou-Jiang Gao, Yu-Chiao Chiu, Lei Li, Yufei Huang

**Affiliations:** 1 Cancer Virology Program, UPMC Hillman Cancer Center, Pittsburgh, PA, USA.; 2 Department of Medicine, University of Pittsburgh, Pittsburgh, PA, USA.; 3 Department of Electrical and Computer Engineering, Swanson School of Engineering, University of Pittsburgh, Pittsburgh, PA, USA.; 4 Intelligent Systems Program, School of Computing and Information, University of Pittsburgh, Pittsburgh, PA, USA.; 5 Electrical and Computer Engineering, University of Texas at Austin, Austin, TX, USA.; 6 Language Technologies Institute, Carnegie Mellon University, Pittsburgh, PA, USA.; 7 Department of Microbiology and Molecular Genetics, University of Pittsburgh School of Medicine, Pittsburgh, PA, USA.; 8 Department of Computational and Systems Biology, University of Pittsburgh School of Medicine, Pittsburgh, PA, USA.; 9 Cancer Therapeutics Program, UPMC Hillman Cancer Center, Pittsburgh, PA, USA.

**Keywords:** AI for Scientific discovery, the EXHYTE cycle, Large language models, Hypothesis generation, Idea generation

## Abstract

Large language models (LLMs) and agent systems are increasingly transforming scientific discovery, driving progress across chemistry, biology, materials science, and physics. Yet most existing work and surveys remain fragmented, focusing on isolated tasks such as idea generation or experiment design without addressing how these components fit within the broader discovery process. To bridge this gap, we introduce the EXHYTE cycle, an iterative framework that formalizes scientific discovery as a sequence of *Exploration*, *Hypothesis generation*, and *Testing*. We assembled a corpus of recent studies, distilled recurring strategies that characterize how AI methods contribute to each EXHYTE substage, and organized the literature accordingly to representative strategies and domain-specific advances. This process-centric perspective unifies diverse methodologies under a single structured workflow, identifies substages that are mature versus underexplored, and reveals complementarities that enable closed-loop discovery systems. It also clarifies the evolving division of labor between human researchers and AI systems, offering a roadmap for developing adaptive, autonomous frameworks for AI-driven scientific discovery. An accompanying website with paper summaries and an LLM-powered interactive survey based on EXHYTE is available at https://webapps.crc.pitt.edu/exhyte/

## 1 Introduction

Recent advances in large language models (LLMs) have enabled a surge of applications in scientific discovery, driving breakthroughs across chemistry [1, 2], biology [3], materials science [4, 5], and physics [6]. Despite this progress, most systems remain focused on isolated tasks, and existing survey papers likewise catalog advances in areas such as idea generation, literature analysis, or experimental design [7, 8]. Although useful, they do not provide a comprehensive view of the full scientific discovery process or how the many AI systems fit together within it.

**Fig. 1:**
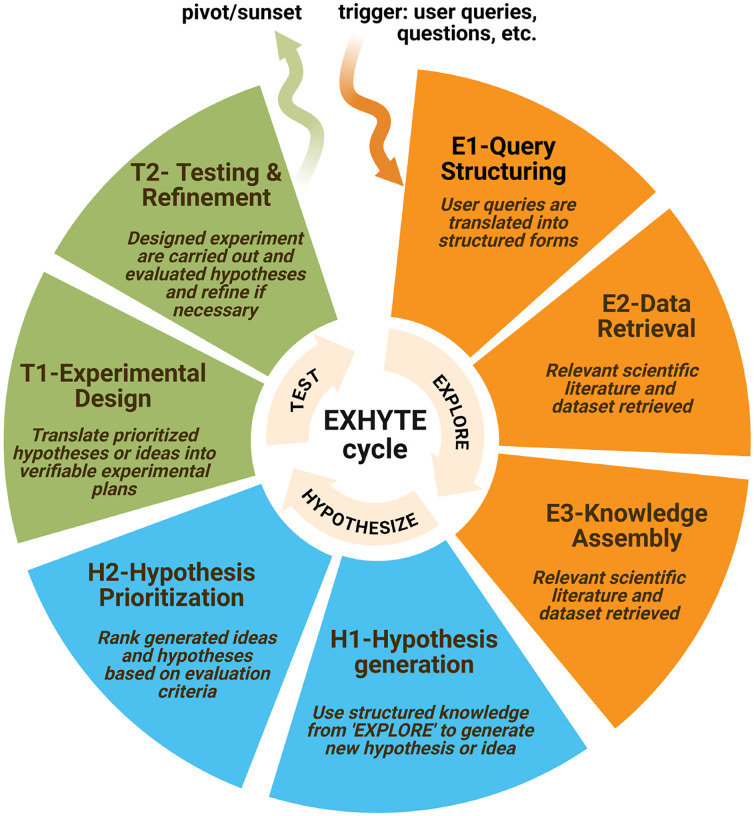
Overview of the EXHYTE cycle for iterative scientific discovery.

Classical scientific inquiry has long been described by the hypothetico-deductive model [9], where a hypothesis is formulated, predictions are derived, and experiments are performed in sequence. Modern research has shifted towards a workflow that is both exploratory and iterative [10]. With advances in high-throughput data, machine learning, and autonomous experimentation, large-scale exploration now serves as the natural starting point, feeding directly into hypothesis generation and repeated testing cycles [11]. To capture this process, we introduce the EXHYTE cycle, which formalizes discovery as a loop of EXploration, HYpothesis generation, and TEsting (Fig. 1). In this cycle, researchers explore existing knowledge and data to identify gaps, generate hypotheses, and test them through targeted experiments, returning to exploration as new evidence emerges. To make the framework more complete, we further divide each stage into substages that reflect the specific activities within each EXHYTE stage. This substage-level view not only mirrors the way research is actually carried out but also provides a finer-grained structure for mapping where AI tools are already contributing and where opportunities remain.

In this paper, we survey existing LLM- and agent-based methods through the lens of the EXHYTE cycle, offering a process-centric perspective that delineates the iterative flow of scientific inquiry and the points of AI intervention. Building on the EXHYTE cycle, we categorize the strategies proposed in the literature for each substage and organize the survey accordingly. This approach has several advantages: (1) it unifies diverse AI advances under a single structured workflow, clarifying how methods from different areas relate to each other; (2) it highlights which substages are well covered and which remain underexplored, pointing to concrete opportunities for innovation; (3) it reveals connections across methods, showing how strategies that target different substages can be combined into closed-loop systems; and (4) it clarifies the division of labor between human researchers and AI systems at each point in the cycle, providing a practical roadmap for building next-generation systems. Grounded in the EXHYTE cycle and extended with a substage-level analysis, our survey provides a coherent and integrative view of how LLMs and agents contribute to scientific discovery and how they can be advanced toward fully autonomous workflows.

## 2 The EXHYTE Cycle: A workflow for data-intensive scientific discovery

We derived the EXHYTE cycle from two complementary sources: recent advances in the philosophy of scientific discovery [9–11], which emphasizes iterative and exploratory models of inquiry, and a systematic review of workflows used in state-of-the-art AI-driven research systems. Across these works, we observed a shared structure in which exploration of large knowledge bases leads naturally to hypothesis generation and targeted testing, forming a self-sustaining loop that integrates human reasoning with computational and experimental processes. EXHYTE formalizes this pattern as an explicit, generalizable framework for data-intensive discovery.

Research typically enters the EXHYTE cycle through a *trigger* (Fig. 1), for example, an open question, an unexpected observation, an anomaly in data, or the introduction of a new measurement technology. Such triggers define a broad area of inquiry but rarely yield immediately testable predictions. The first stage, EXPLORE, therefore seeks to organize what is known and expose what remains uncertain.

**EXPLORE** aims to assemble and structure knowledge so that knowledge gaps become explicit and actionable. This stage consists of three substages:

- **E1: Query structuring** translates research questions into machine-actionable forms (e.g., key phrases, controlled vocabularies, domain schemas) to guide retrieval.
- **E2: Data retrieval** collects literature, code, and structured datasets from scientific repositories and APIs according to the structured query plan in E1.
- **E3: Knowledge assembly** normalizes and integrates retrieved data into representations such as embeddings, graphs, or feature tables and summarizes evidence to surface explicit knowledge gaps.

The output of EXPLORE is a structured knowledge base and a set of defined knowledge gaps that motivate the next stage.

**HYPOTHESIZE** transforms knowledge gaps into ideas or specific, testable claims through two substages:

- **H1: Hypothesis/idea generation** formulates candidate mechanisms or interventions using approaches such as literature-grounded reasoning or data-driven strategies.
- **H2: Hypothesis/idea prioritization** evaluates these candidates for novelty, plausibility, feasibility and expected impact using quantitative metrics or expert and LLM-based assessments.

The outcome of this stage is a prioritized list of hypotheses with clear rationales and success criteria, ready for empirical validation.

**TEST** translates hypotheses into executable experiments and closes the feedback loop in two substages:

- **T1: Experimental design** specifies objectives, protocols, datasets/materials, and measurable outcomes, yielding executable experimental or computational plans.
- **T2: Testing and refinement** performs *in silico*, *in vitro* or *in vivo* experiments and analyzes the results to confirm, refine, or reject hypotheses.

Results from TEST feed back into the cycle through one of the three paths. When the findings generally support the hypothesis but leave important uncertainties, the process proceeds through refinement by adjusting parameters, recording additional variables, or refining the experimental design while maintaining the same mechanistic focus. When evidence contradicts the hypothesis, the cycle pivots, preserving the original knowledge gap but exploring alternative explanations or mechanisms in the next iteration. In contrast, when success criteria are fully met and the research question is resolved, the inquiry sunsets, concluding the current line of investigation rather than looping back to further exploration. Together, these outcomes ensure that EXHYTE functions as a self-correcting, evidence-driven process, capable of continuous adaptation while allowing closure when discovery goals are achieved.

## 3 AI Methods for the EXHYTE Cycle

To construct the survey corpus, we focused on the literature published between 2018 and July 2025 to capture recent advances in LLMs and agent systems for scientific discovery. Articles were retrieved through keyword searches on the arXiv and Semantic Scholar APIs, using queries such as LLM, idea generation, hypothesis generation, scientific discovery, experiment design, material discovery, molecular design, drug discovery, and biomedical discovery. Retrieved results were then manually filtered to remove irrelevant articles and ensure inclusion of high-quality papers from major conferences and journals. The final corpus comprises 83 papers spanning diverse disciplines.

**Fig. 2:**
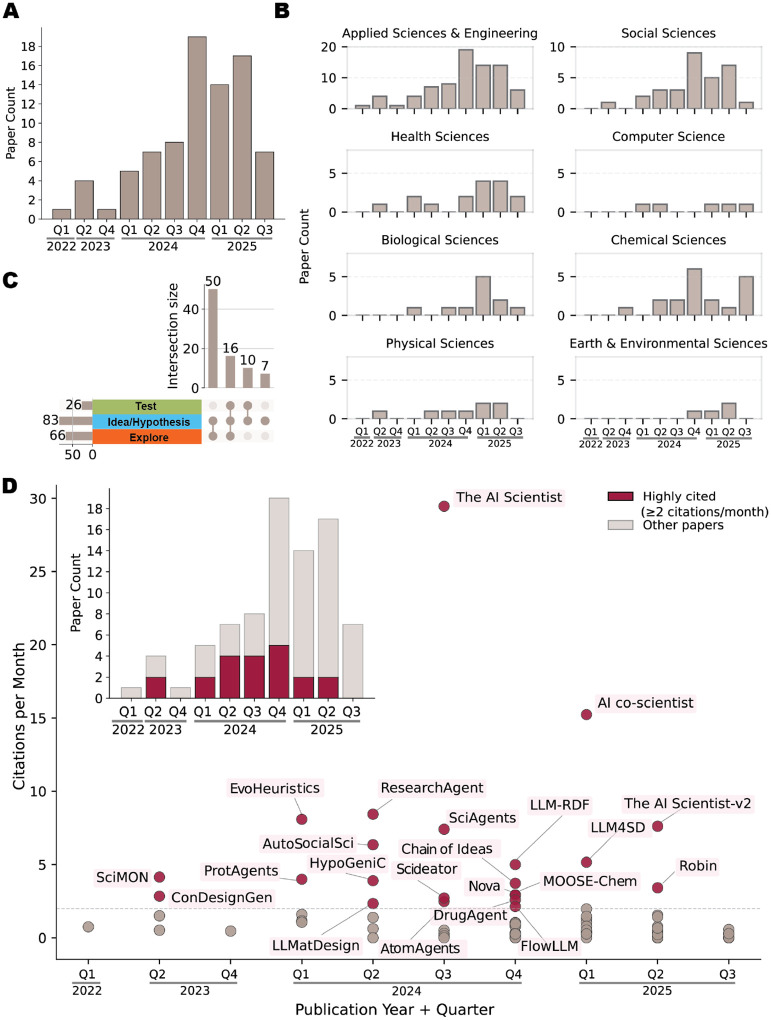
Temporal and thematic analysis of publications. (A) Quarterly count of publications over time. The bar height represents the number of papers published in each quarter. (B) Quarterly distribution of publications by subject area. Bars indicate the number of papers in each subject area per quarter. (C) Upset plot showing the number of publications covering different stages of the EXHYTE cycle (Explore, Idea/Hypothesis, Test). Bars represent the number of papers in each combination of stages. (D) Scatter plot of highly cited papers (red dots), with citations per month on the y-axis and quarter/year on the x-axis. Highly cited papers are defined as those receiving ≥ 2 citations per month. The red bars in the overlaid bar plot indicates the number of highly cited papers per quarter.

The publication volume in this area surged between 2023 and 2024 and continued to rise sharply in 2025 (Fig. 2A), reflecting both growing cross-domain interest and rapid methodological progress in applying AI to scientific discovery. In addition, across subject areas, most papers come from Applied Sciences and Engineering, followed by Social, Chemical, and Biological Sciences (Fig. 2B). The distribution of highly cited papers over time (Fig. 2D) reveals that several works have gained meaningful impact within the research community. Based on our systematic review of the corpus, 50 papers progress beyond exploration to the HYPOTHESIZE stage(idea/hypothesis generation), yet only 16 continue to the TEST stage (Fig. 2C). This disparity highlights a persistent disconnect between hypothesis generation and empirical verification in contemporary AI-assisted scientific discovery. Together, these patterns reveal a field that is expanding rapidly in scope and influence while still exhibiting uneven progress across stages of the discovery workflow, with substantial room for methodological advances that bridge hypothesis generation and empirical testing.

To systematically analyze and organize this rapidly expanding body of work, we identified recurring AI strategies that address challenges in each substage of the EXHYTE cycle (see Supplementary file). Organizing the literature around these strategies moves beyond task- or domain-specific taxonomies, providing a structured view of how AI methods operate across the EXHYTE workflow. In the following sections, we present these strategies substage by substage, defining core patterns, summarizing representative studies and domain-specific variants, and highlighting how each approach is implemented and supported by empirical evidence.

### 3.1 Inputs to the EXHYTE Cycle

In automated scientific discovery systems, user input ranges from natural language queries about research topics to domain-specific research papers or datasets, playing a central role in aligning system behavior with specific goals. High-level research directions, such as workshop themes, set the scope for the automated scientific workflow. For example, the ICLR 2025 workshop “I Can’t Believe It’s Not Better” (ICBINB) focused on real-world pitfalls, challenges, and negative or inconclusive results in deep learning, a perspective incorporated in AI-Scientist-v2 [12]. Similarly, focused problem statements, such as “How can we improve the energy efficiency of AI models?” (e.g., SCI-IDEA [13]), guide idea and hypothesis generation. Extending this idea, IRIS [14] formalizes user input as research goals that are explicitly transformed into a problem and its motivation/context. For example, the research goal “To improve the balance between quality and diversity in text generation (Problem) by addressing the limitations of existing sampling methods like top-p (nucleus sampling), which often produce incoherent or repetitive outputs at higher temperatures (motivation/context)”. Structured, domain-specific inputs similarly allow users to express goals and constraints in scientific formats. For example, LLMatDesign [5] begins with user-specified chemical compositions and target properties for candidate materials, while AutoMAT [15] accepts user-specified targets such as yield strength, density, or elemental cost constraints. In drug discovery, systems such as [16] operate on query molecules represented as SMILES strings and PiFlow [17] integrates foundational scientific laws or expert-defined principles to guide search.

Beyond explicit goal specification, user-provided resources such as research papers and datasets function as empirical and contextual inputs. Systems like ResearchAgent [18], CodeScientist [19], and HypER [20] leverage literature retrieval and idea grounding. CodeScientist [19], for example, summarizes vetted code blocks to ensure that generated research ideas are practical, implementable, and executable. Similarly, datasets provide an empirical foundation for discovery. DrugMCTS [21], for example, employs molecule–protein interaction datasets such as DrugBank [22] and KIBA [23] to support drug discovery tasks.

Together, these examples highlight the pivotal role of user input, whether conveyed through language, structured scientific specifications, or empirical data, in steering automated discovery workflows across diverse scientific domains.

### 3.2 The Explore Stage

#### 3.2.1 E1: Query Structuring

**Fig. 3:**
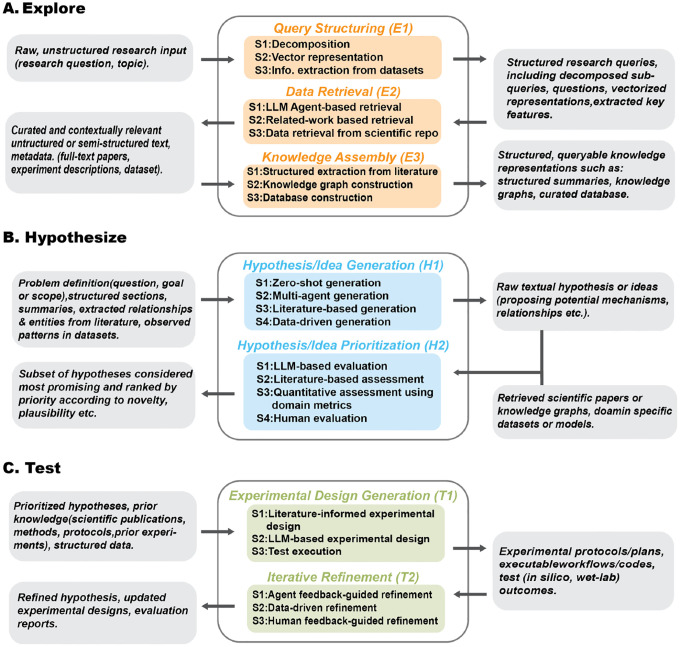
Inputs and outputs associated with each substage of the EXHYTE workflow.

**Fig. 4:**
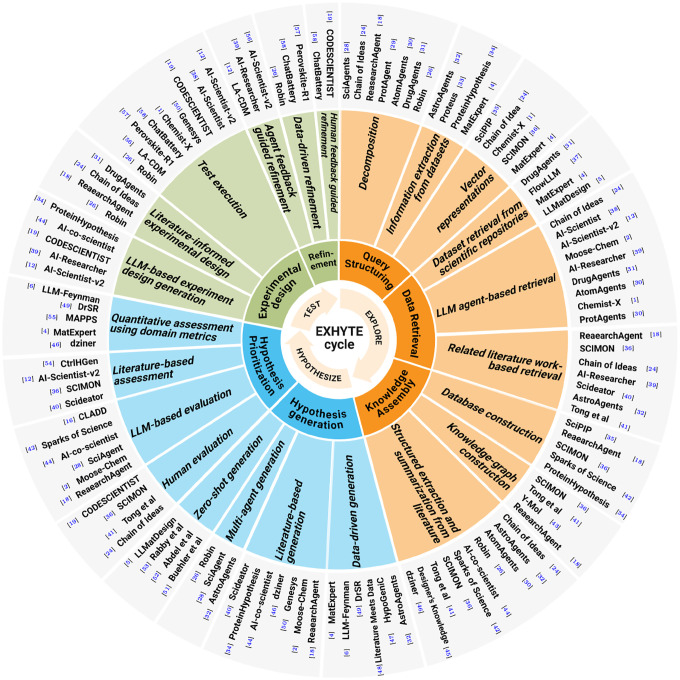
AI methods for the EXHYTE Cycle and substages.

User query structuring is the first step in the Explore Stage, where it transforms raw, unstructured research inputs into actionable representations. This process employs complementary strategies including decomposition, vector representation, and information extraction from datasets to enable targeted exploration across literature and data (Fig. 3A and 4).

##### E1-S1: Decomposition.

Decomposition transforms complex research queries into tractable sub-queries/questions or tasks that enable focused literature/dataset retrieval, reasoning, and solution design using LLM. Different systems operationalize decomposition at various levels. Chain of Ideas [24] decomposes initial research topic into multiple sub-queries that capture diverse perspectives on the same problem., SCI-IDEA [13] extracts key-phrases that represent the underlying research focus of a scientist’s query, Assay2Mol [25] identifies keywords from user-provided text descriptions of target proteins or phenotypes, Robin [26] formulates a series of general questions based on the name of the target disease that the scientist inputs into the system, DrugLLM [27] decomposes molecules into structural groups to facilitate reasoning about chemical properties.

Building on this principle, several systems employ LLM-based agents to decompose complex user inputs, whether research queries or design tasks, into smaller, manageable sub-tasks and to generate detailed, step-by-step workflow plans. General-purpose frameworks such as ResearchAgent [18], AI-Researcher [39], SciAgent [28], Zhou et al. [55] and domain specific system such as ProtAgent(protein design) [29], AtomAgents(alloy design) [30], MatExpert(material discovery) [4],DrugAgents(drug discovery) [31] all follow similar decomposition pipeline.

##### E1-S2: Vector representations.

Encoding user inputs as vector representations enables efficient retrieval, semantic similarity search, and context-aware reasoning. Frameworks such as Chain of Ideas [24], SciPIP [35], SCI-IDEA [13], SCIMON [36], IRIS [14] transform research queries, questions, background knowledge into embeddings to support semantic retrieval of relevant scientific literature.

In domain-specific applications, Chemist-X [1] encodes molecules as SMILES strings to retrieve reaction conditions for target products, while DrugMCTS [21] identifies structurally similar molecules using vectorized molecular representations. Assay2Mol [25] embeds protein target descriptions and phenotypic data for BioAssay retrieval, and MatExpert [4] employs dual encoders with one for property descriptions and another for structural representations to improve reference material retrieval. Collectively, these systems underscore the pivotal role of vector representations in enabling semantically rich, machine-interpretable forms of knowledge that drive efficient and context-aware scientific discovery

##### E1-S3: Information Extraction from Datasets.

Extracting key features and annotations from structured datasets enables automated systems to generate empirical inputs for downstream tasks such as model training, hypothesis formulation, and idea generation. AstroAgents [32], for instance, employs a *Data Analyst Agent* to identify polycyclic aromatic hydrocarbon (PAH) patterns from user-provided mass spectrometry data, thereby generating testable hypotheses in astrobiology. Proteus [33] integrates bioinformatics tools to uncover biological relationships through statistical analyses, while Qu et al. [33] further identify pairs of biological entities (e.g., protein, clinic) to define research directions from multi-omics datasets. ProteinHypothesis [34] retrieves protein sequence descriptions, structural features, and physicochemical properties to inform LLMs about sequence–structure–function relationships during hypothesis generation. In drug discovery, DrugMCTS [21] extracts molecular properties and annotations from DrugBank [22] and KIBA [23], while MatExpert [4] extracts chemical formulas and physical properties (e.g., formation energy, elemental composition) from databases such as NOMAD [59] and Materials Project [60] to generate candidate crystal structures. LLM-Feynman [6] automates feature selection and iterative refinement to construct high-quality feature sets, which are subsequently used in symbolic regression to derive interpretable physical equations. Together, these systems illustrate how information extraction transforms raw datasets into structured, semantically meaningful inputs that drive empirical reasoning and hypothesis development across scientific domains.

#### 3.2.2 E2: Data Retrieval

Retrieving literature and datasets is a foundational step in scientific discovery, ensuring that exploration remains grounded in comprehensive and relevant knowledge. Guided by user inputs, this E2 step compiles scientific publications and datasets that support informed hypothesis exploration and validation. We identified three key strategies that capture how systems retrieve information through LLM-based agents, related-work networks, and scientific repositories, respectively (Fig. 3A and 4).

##### E2-S1: LLM Agent–Based Retrieval.

LLM agents now routinely query and curate literature and datasets. General-purpose frameworks such as Chain of Ideas [24] and Graph of AI Ideas [61] use LLMs to generate multiple research queries from an initial topic, capturing diverse conceptual angles. SciAgents [28] and AI-Scientist [38], along with AI-Scientist-v2 [12], retrieve literature via the Semantic Scholar API for novelty checks and evidence gathering.

LLM agents are also tasked with prioritizing the most relevant papers. IRIS [14] introduces a retrieval agent that issues targeted queries answered by the specialized “AI2 Scholar QA” API. Moose-Chem [2] generates titles from background papers to retrieve inspirational works. AI-Researcher [39] designs a “Knowledge Acquisition Agent” that, given reference papers, retrieves additional papers and code repositories from scientific databases.

In domain-specific settings, retrieval is coupled with scientific reasoning. DrugAgent [31] employs an “Instructor Agent” to query domain libraries and translate abstract ideas into executable code via domain-specific examples. AtomAgents [30] integrates a specialized knowledge-retrieval tool that extracts material properties directly from published papers. Chemist-X [1] produces executable code to interrogate repositories such as PubChem for chemical literature and molecular datasets, while ProtAgents [29] retrieves specific data points (e.g., Protein Data Bank (PDB) identifiers) for protein design. In materials discovery, AutoMAT [15] uses LLMs, together with user-defined property targets and constraints, to consult reference handbooks (e.g., titanium alloy databases) and identify candidates meeting elemental criteria.

##### E2-S2: Related-Work Based Retrieval.

Citation networks and semantic similarity are widely used to link related works and surface new directions. ResearchAgent [18], SCIMON [36], HypER [20], and Chain of Ideas [24] expand from an anchor paper by retrieving both citing and cited works to capture the evolution of research themes; HypER further adds LLM-based relevancy scoring. AI-Researcher [39] incorporates multi-step reference selection for each target paper, including citation-pattern analysis (frequency), context analysis (influence on methods/theory/experiments), evidence collection (textual support), and an integrated impact score. SciPIP [35] combines keyword, co-occurrence, and semantics-based retrieval, then applies a cluster-based selection of representative papers. SCI-IDEA [13] queries academic sources using key phrases drawn from a researcher’s work and similar literature. Scideator [40] retrieves multiple analogs per source paper, uses an LLM to generate “purpose–mechanism” pairs, and then issues queries per pair to find additional relevant papers. Domain-focused approaches tailor queries to specialized terms. For example, ProteinHypothesis [34] uses domain-specific keywords (e.g., “Protein Science”) to retrieve metadata (titles, abstracts, links); AstroAgents [32] selects relevant papers and books to provide astrobiology context; and Tong et al. [41] require the presence of terms such as “psychol,” “clin psychol,” and “biol psychol” in titles or abstracts for psychology/neuroscience. Manual and semi-automatic curation complements these methods via direct selection from leading databases (e.g., Sparks of Science [42], Perovskite-R1 [57], Budget AI Researcher [62], Lee et al. [16], Quilodrán-Casas et al. [63], Cheng et al. [50], Ciucua et al. [64]).

##### E2-S3: Dataset Retrieval from Scientific Repositories.

Datasets provide the empirical foundation for analysis, model training, and validation. In drug discovery, DrugMCTS [21] queries databases to retrieve molecules structurally similar to a query compound, associated protein interactors, and binding-pocket information parsed from Protein Data Bank (PDB) files to improve LLM interpretability. DrugAgent [31] retrieves biological datasets from the Therapeutics Data Commons (TDC), which offers structured, AI-ready datasets and benchmarks across drug development stages. In materials discovery, FlowLLM [37] retrieves inorganic crystalline materials from the Materials Project for LLM fine-tuning, and Perovskite-R1 [57] assembles drug-like compounds from a comprehensive library to enhance generalization across chemical space. MatExpert [4] and LLMatDesign [5] access NOMAD [59] and the Materials Project [60] for reference materials used in training, evaluation, and modification workflows. Zhou et al. [55] retrieve crystal structures with compositions or reduced formulas similar to a query from structural databases to identify promising crystal prototypes.

#### 3.2.3 E3: Knowledge Assembly

The knowledge-assembly stage transforms unstructured scientific text into structured, queryable representations that enable efficient reasoning during discovery. The existing literature typically employs three strategies: structured extraction and summarization from literature, knowledge-graph construction, and database construction for large-scale retrieval and synthesis (Fig. 3A and 4).

##### E3-S1: Structured extraction and summarization from literature

To make literature readily usable for idea and hypothesis generation, systems extract and summarize standardized sections (e.g., titles, abstracts, methods, results). SciPIP [35], Chain of Ideas [24], SCI-IDEA [13], and AstroAgents [32] implement pipelines that segment papers into consistent fields. Papers like SciPIP [35], AtomAgents [30], Robin [26], Literature Meets Data [48], AI-co-scientist [44] and IRIS [14] further produce concise synopses of key findings. Sparks of Science [42] uses LLMs to extract each paper’s conventional assumption and innovative approach alongside a concise insight summary from abstracts. Several systems including SCI-IDEA [13], Scideator [40], SCIMON [36], and Chen et al. [65] extract facets such as objectives, methods, evaluation, and future work. Domain-tailored extraction includes causal relations in psychology (Tong et al. [41]), question–answer pairs for Perovskite precursors (Perovskite-R1 [57]), frequent-topic mining to guide retrieval (Lee et al. [62]), structured tables of target properties, related structures, and linking mechanisms (Beyond Designer’s Knowledge [45]), parsing mechanical properties and compositions to propose alloys (AutoMAT [15]), and sentence-level evidence selection for property-optimization queries (dziner [46]).

##### E3-S2: Knowledge graph construction.

Knowledge graphs (KGs) encode entities and relations to support structured reasoning and discovery. SCIMON [36] represents tasks, methods, materials, and metrics as nodes, with “used-for” edges to ground generated ideas. Tong et al. [41] construct causal KGs in psychology, where (cause, effect) pairs are directed nodes with attributes (interpretations) and relation types (causal vs. correlational). Y-Mol [43] builds a biomedical KG with interactions among drugs, genes, and diseases (Drug–Target, Drug–Drug, and Disease–Gene). ResearchAgent [18] assembles an entity-centric store from co-occurrences in literature to expose cross-domain connections (topics, keywords, individuals, events). SciPIP [35] links papers to LLM-extracted keywords in a paper–keyword graph for efficient keyword-based retrieval. Graph of AI Ideas [61] constructs a paper-level graph using structured quadruples *(paper, citation position, citation semantics, paper)*, where citation position captures reference importance and citation semantics defines the purpose of each citation.

##### E3-S3: Database construction.

Curated databases consolidate heterogeneous resources into structured repositories for retrieval, analysis, and synthesis. SciPIP [35] builds a literature database from top venues over the past decade, storing key sections and re-summarizing each paper into a quintuple (keywords, background, ideas, concise methods, core references), with background/ideas encoded as embeddings. ResearchAgent [18] extracts entities from titles/abstracts (e.g., post-2023 Semantic Scholar) and encodes their co-occurrences in a sparse matrix. HypER [20] compiles temporal reasoning-chain datasets linked to related papers for effective reasoning over scientific literature. SCIMON [36] builds corpora from the ACL Anthology and PubMed, organizing the papers with extracted (Background, Target) pairs, seed terms, and their relations. Sparks of Science [42] constructs the “HypoGen” dataset by extracting assumptions, innovations, and core insights from abstracts. ProteinHypothesis [34], Lee et al. [62], and Cheng et al. [50] store vector embeddings of papers from arXiv and major conferences (e.g., NeurIPS, ICLR) to support scalable retrieval.

### 3.3 The Hypothesize Stage

#### 3.3.1 H1: Hypothesis/Idea Generation

Hypothesis and idea generation mark the transition from structured knowledge to novel discovery, synthesizing literature, data, and reasoning strategies into creative, testable hypotheses. We identify four strategies commonly employed in the literature, including zero-shot generation, multi-agent generation, literature-based generation, and data-driven generation (Fig. 3B and 4).

##### H1-S1: Zero-shot generation.

Zero-shot generation relies on the internal knowledge encoded in LLMs, thus enabling idea/hypothesis creation without explicit external context—useful when domain-specific training data are limited. Frameworks illustrating this approach include LLMatDesign [5], Buehler et al. [51], Tang et al. [66], Yin et al. [67], Bystronski et al. [68], Abdel et al. [52], Rabby et al. [53], Guo et al. [69], Pu et al. [17], Liu et al. [70], and Manning et al. [71].

##### H1-S2: Multi-agent generation.

Multi-agent frameworks build specialized agents, coordinated under an orchestrator agent to produce and refine hypotheses. AstroAgents [32] employs a hierarchical design, where a ‘Data Analysis Agent’ first uncovers key patterns and trends and a ‘Planner Agent’ delegates distinct data segments to multiple ‘Scientist Agents’ for in-depth exploration and investigation; finally each scientist operates within its assigned domain to produce novel hypotheses, which are consolidated by an ‘Accumulator Agent’ that deduplicates and integrates the results.

In the KG-grounded SciAgents [28], an Assistant Agent drafts an initial subgraph to contextualize the idea, an Ontologist Agent defines nodes and interprets edges, and two Scientist Agents iteratively draft, elaborate, and critique detailed hypotheses. Robin [26] specializes in biomedicine and designed an agent ‘Crow’ to identify causal disease mechanisms and suitable *in vitro* models and another agent ‘Falcon’ to conduct deep literature review to evaluate therapeutic candidates. In chemical discovery, DrugMCTS [21] coordinates multiple sequential agents to incrementally build reasoning paths for protein target identification, where a ‘Molecule-Analysis’ Agent produces natural-language molecular reports, a ‘Molecule-Selection Agent’ filters candidates, an ‘Interaction-Analysis’ Agent explores molecule–protein interactions, and a ‘Decision’ Agent synthesizes upstream findings to predict promising targets.

##### H1-S3: Literature-based generation.

Literature-driven approaches retrieve, synthesize, and recombine knowledge from scientific texts to propose novel hypotheses. ResearchAgent [18] and Moose-Chem [2] provide titles/abstracts of various related papers as context for LLM. Genesys [50] formulates new LLM design ideas by modifying parent designs from the Knowledge Engine (KE), which integrates the curated literature with code and external sources. Similarly, dziner [46] generates modifications based on retrieved material-design guidelines mined from unstructured text (e.g. scientific literature and the Internet). Another common strategy is summarization-based synthesis, where relevant sections are distilled and restructured before prompting the LLM (e.g., AI-co-scientist [44], SciPIP [35], ProteinHypothesis [34], Perovskite-R1 [57]). Another approach is facet recombination, in which facets (e.g. purpose and mechanism) are recombined to surface gaps and generate ideas. For example, Scideator [40] constructs analogies from purpose–mechanism pairs across papers; SCI-IDEA [13] analyzes similar facets to detect patterns, inconsistencies, and unexplored areas; Liu et al. [45] and Lee et al. [62] synthesize novel interdependencies among mechanisms or topics not explicitly linked in the source literature; and CODESCIENTIST [19] explores combinatorial idea generation via genetic operators (e.g., crossover, mutation). A complementary line of work is a reasoning-chain–based generation, which reconstructs explanatory pathways from the noisy/fragmented literature. HypER [20], Sparks of Science [42], and Graph of AI Ideas [61] fall into this line and identify valid chains in the literature graphs and use them as scaffolds for evidence-grounded hypotheses. Finally, KGs are proposed to structure scientific concepts and relations for systematic hypothesis generation. For example, Tong et al. [41] employ link prediction algorithms on causal knowledge graphs to identify high-probability causal relationships among previously unconnected concepts.; Lee et al. [16] integrate biochemical drug–drug relationships from a KG to interpret the network structure, then pass these summaries to a prediction agent; Gao et al. [54] generate plausible logical hypotheses from KG entity sets.

##### H1-S4: Data-driven generation.

Structured datasets provide a complementary source, where data instances and patterns can guide hypothesis formation. One group of systems detects patterns and supplies them as context to the LLM. In AstroAgents [32], a ‘Data Analyst’ Agent examines mass spectrometry to uncover PAH (Polycyclic Aromatic Hydrocarbons) distributions and alkylation patterns, highlighting unexpected findings. ProteinHypothesis [34] analyzes sequence motifs, secondary-structure correlations, and functional-site patterns from experimental data. Proteus [33] runs targeted statistical analyses over multi-omics data using bioinformatics tools, recording quantified relationships between biological entities (e.g. proteins, transcripts, variants) and clinical features (e.g. survival time, tumor stage) as edges in a ‘Conclusion Graph’ that then grounds LLM hypothesis generation. Another line seeds the LLM with few-shot data instances: HypoGeniC [47], Literature Meets Data [48], and Hypotheses for Inductive Reasoning [72] provide small sets of (input, label) pairs to induce initial hypotheses. In DrSR [49], ChatSR [73], and LLM-Feynman [6], observational pairs guide LLM in analyzing structural relationships, which are then used by symbolic regression to produce interpretable formulas. Zheng et al. [74] discover novel molecular patterns using structural/chemical features and then use them to predict molecular properties. Liu et al. [75] retrieve domain-specific drug data as demonstrations for LLM to perform drug-editing tasks. In material, MAPPS [55] queries structural databases for crystal prototypes with compositions/reduced formulas similar to a query, using them to generate initial candidate structures; MatExpert [4] fine-tunes a small LLM on curated transition pathways and target materials from NOMAD to generate plausible transition pathways. In healthcare and clinical design, Zhu et al. [76] and Bani et al. [56] fine-tune LLMs on patent–title pairs or clinical datasets containing various clinical scenarios and ground-truth diagnoses to enable new design ideas and hypotheses.

#### 3.3.2 H2: Hypothesis or Idea Prioritization

Hypothesis prioritization focuses analytical and computational resources on the most promising ideas by evaluating their novelty, plausibility, feasibility, and potential impact. We identify four common strategies: LLM-based evaluation, literature-based assessment, quantitative assessment using domain metrics, and human evaluation (Fig. 3B and 4).

##### H2-S1: LLM-based evaluation.

LLM agents can act as evaluators, ranking ideas/hypotheses on clarity, feasibility, novelty, plausibility, consistency, and impact. Exemples include ResearchAgent [18],AstroAgents [32], SciAgents [28], AI-co-scientist [44], Sparks of Science [42], Moose-chem [2], Tong et al [41], PROTEUS [33], Rabby et al [53], ProteinHypothesis [34]. Robin [26] uses an LLM judge to rank drug candidates by scientific rationale, pharmacological profile, and methodology of the supporting literature. AstroAgents [32] uses a critic agent to assess the alignment between hypotheses and observational data. CLADD [16] evaluates drugs by predicting its toxicity from molecule’s chemical representation(SMILES), infers likely protein targets (mechanism of action) from LLM agent generated comprehensive report on query molecule, and property categorization performance of molecules based on generated textual description concatenated with its SMILES representation. DrugMCTS [21] extends this by integrating LLM-based reward functions within a Monte Carlo Tree Search framework to score molecular–protein interactions using structural (binding pocket) and literature-derived contextual evidence. ChatBattery [58] uses a ‘Search’ Agent to verify novelty by querying the Materials Project database and a ‘Retrieval’ Agent to identify similar lithium-based compounds from the ICSD, guiding subsequent hypothesis revisions.

Liu et al [45] evaluate materials design hypotheses based on the alignment between the stated materials design goals and the content of the hypothesis. LLM-Feynman [6] employs an LLM to assign ‘interpretability score’ to its own generated formulas, quantifying their physical and chemical meaning and ProtAgents [29] assess success in generating proteins with desired secondary structures.

##### H2-S2: Literature-based assessment.

To ensure that generated hypotheses are both novel and scientifically grounded, many systems incorporate literature-based assessment as a critical step. This process involves retrieving relevant prior work, evaluating semantic similarity, and determining whether the proposed idea contributes to new insights. Systems such as Scideator [40], HypER [20], SCIMON [36], AI-scientist [38], AI-Scientist-v2 [12], AstroAgents [32], Graph of AI Ideas [61], SCI-IDEA [13], SciAgents [28], Budget AI Researcher [62], Cheng et al [50], Lee et al [62] deploy LLM-based ‘Novelty Checker’, which assesses the idea’s novelty against relevant papers. HypER [20] evaluates whether the rationale and hypothesis track the logic and chronology of literature chains. CtrlHGen [54] measures the alignment between hypothesis conclusions and the observed entities in the knowledge graph using multiple similarity metrics. These metrics are incorporated into the reward function that guides hypothesis generation.

##### H2-S3: Quantitative Assessment Using Domain Metrics.

Quantitative evaluation using domain-specific scientific metrics and computational models provides an objective measure of hypothesis. dziner [46] calculate ‘synthesizability’ (e.g. synthetic accessibility score, quantitative estimate of drug-likeness (QED score)) using PubChem-scale molecule dataset and specialized tools. The effectiveness of a proposed modification is then evaluated with domain-expert predictive models, and the LLM ultimately decides whether to accept or reject the modification based on these quantitative results. Matexpert [4] evaluates generated material structural validity, chemical feasibility and stability using quantitative and physics-informed metrics. ChatBattery [58] verifies the stability and properties of the top-ranked cathode candidates using a machine learning surrogate model(MACE-MP) that predicts the energies and forces of the sampled structures. MAPPS [55] evaluates crystal structures for structural quality, thermodynamic stability, novelty, and accuracy. Liu et al [75] compare edited molecules with desired properties, quantify binding affinity for peptide editing and secondary structural content for protein secondary structure editing relative to desired targets. LLMatdesign [5] predicts properties via a Machine Learning Property Predictor (MLPP) after a Machine-Learning-Force-Field (MLFF) based relaxation of modified material structures, declaring success when the predicted properties of the new material align with target specifications. In symbolic reasoning and equation discovery, DrSR [49] and ChatSR [73] iteratively improve model output by assessing generated equations using numerical fitness scores and domain performance metrics. LLM-Feynman [6] provides a comprehensive quantitative evaluation framework using the Coefficient of Determination and Mean Absolute Error (MAE) for regression tasks, and accuracy, precision, recall, F1 score, and cross-entropy loss for classification-based symbolic modeling.

##### H2-S4: Human evaluation.

Expert review complements automated scoring with contextual judgment and domain nuance. Chain of Ideas [24], Tong et al [41], AstroAgents [32], SciPIP [35], SciMON [36], CODESCIENTIST [19], SCI-IDEA [13], Literature Meets Data [48], ChatBattery [58], Rabby et al [53] and HypER [20] incorporate expert feedback to refine, rank, or filter hypotheses.

### 3.4 The Test Stage

#### 3.4.1 T1: Experimental Design Generation

Experimental design generation translates top-ranked ideas and hypotheses into verifiable experimental plans, creating a systematic path to validate or refute scientific propositions. It comprises three common strategies: literature-informed design, LL-based design generation, and test execution (Fig. 3C and 4).

##### T1-S1: Literature-informed experimental design.

This strategy leverages prior studies and domain references to ground experimental plans in established scientific practices while enabling innovation. Robin [26] selects top-ranked ‘in vitro’ models from the literature review to define therapeutic testing strategies and generates concise experimental outlines. ResearchAgent [18] synthesizes problem information, methods, and existing designs from the literature into new protocols that emphasizing clarity, robustness, reproducibility, validity, and feasibility. Chain of Ideas [24] uses the few-shot prompting with examples of prior experiments, together with user-provided ideas and key entities, to guide design generation. Similarly, DrugAgent [31] constructs executable code from curated documentation, compiled from widely used libraries and models from the literature. Together, these approaches ensure the design evidence-based while adaptable to novel scientific contexts.

##### T1-S2: LLM-based experiment design generation.

Beyond literature grounding, systems increasingly rely on LLM reasoning to autonomously generate experimental designs, often producing executable workflows or code for validation. AI-Scientist-v2 [12] employs ‘Agentic Tree Search algorithm’ to systematically explore hypotheses during the experimentation phase. For each node in the experimental pathway, an LLM proposes a concrete experimentation plan and the Python code to implement it. Similarly, AI-researcher [39], Lee et al [72], Lui et al [70], Cheng et al [50] transform research analyses and development plans into executable code via LLM agents. In physics-based discovery, Zhou et al [55] invoke tools from a physics toolbox to translate workflow steps into executable, physically grounded Python code. CODESCIENTIST [19] uses a ‘planer agent’ that ingests the idea, expert comments, and a codeblock library to produce a concrete plan and required codeblocks. AI-co-scientist [44] features a ‘Generation Agent’ that proposes experimental protocols for downstream validations. In biomedical experimental setting, ProteinHypothesis [34] assesses the experimental feasibility of hypotheses and maps the hypotheses to laboratory techniques, while Chemist-X [1] generates control scripts for an agent to operate wet-lab platforms for validation.

##### T1-S3: Test execution.

This strategy validates LLM-generated hypotheses via *in silico* or wet-lab experiments, closing the discovery loop by determining the real-world feasibility and accuracy of these hypotheses. In Robin [26], the experimental strategies are proposed for validating therapeutic candidates by identifying a relevant disease mechanism and *in vitro* models and executed by human researchers in an iterative labin-the-loop framework. In clinical hypothesis refinement, LA-CDM [56] uses a ‘decision agent’ to request additional diagnostic tests to reduce uncertainty. Perovskite-R1 [57] implements recommended protocols and fabricates complete solar cell devices to assess the efficacy of the selected additives. ChatBattery [58] synthesizes and tests cathode candidates through wet-lab experiments. Chemist-X [1] validates generated reaction conditions through fully automated wet-lab experiments under LLM supervision. Abdel et al [52] test LLM-proposed drug combinations in the lab.

Execution also includes computational validation. Tang et al [66] apply regression-based statistical models to test hypotheses about LLM’s explanatory power for societal outcomes. Genesys [50] evaluates the proposed language model design by verifying code correctness, conducting training-time experiments, and measuring success via a multi-scale fitness function. CODESCIENTIST [19] follows an iterative generate–execute–reflect debugging loop, while AI-Scientist [39] and AI-Scientist-v2 [12] test LLM-generated experiment prototypes by running associated Python code to test conceptual validity.

#### 3.4.2 T2: Iterative refinement

Iterative refinement improves the quality and feasibility of hypotheses, ideas, and experimental designs by incorporating feedback from agents, data, and experts, ensuring responsive to new information and evaluations. There are three common strategies: agent feedback–guided refinement, data-driven refinement, and human feedback–guided refinement (Fig. 3C and 4).

##### T2-S1: Agent feedback–guided refinement.

Systems employ feedback loops in which LLM-based agents iteratively revise hypotheses, code, and designs to improve feasibility and correctness. AI-Scientist-v2 [12] refines algorithm nodes through agent feedback by evaluating parent nodes’ outcome, where buggy parents trigger debugging using clind nodes, while non-buggy parents prompt experimental refinement. Key metrics (e.g., loss values, training data) are logged and visualized and a Vision–Language Model (VLM) then critiques plots for label clarity, legend completeness, and data accuracy. Nodes flagged by the VLM are marked buggy, whereas validated nodes are retained, enabling continuous, feedback-driven improvement. Similarly, AI-researcher [39] utilizes an ‘Advisor’ Agent to propose actionable refinement spanning implementation details, validation studies, visualizations, and comparative analyses. In clinical settings, the ‘Hypothesis’ Agent in LA-CDM [56] updates diagnoses and confidence estimates as patient-state information evolves, repeating until a final diagnosis is produced.

##### T2-S2: Data-driven refinement.

Data-driven refinement keeps hypotheses, models, and experimental plans empirically grounded by updating them with new computational and experimental evidence. In Robin [26], human scientists upload raw or semi-processed data (e.g., flow cytometry, RNA-seq data), which are processed by the ‘Finch’ analysis agent with the requested analysis to generate statistical tests, differential gene expression plots. Robin also distills actionable insights from the analysis results and proposes follow-up assays. Fully automated systems such as Chemist-X [1] execute LLM-generated reaction conditions with closed-loop wet-lab experiments and quantify reaction outcomes (e.g., the product yield using High-Performance Liquid Chromatography). Abdel et al. [52] validate LLM-generated drug combinations experimentally and compute Highest Single Agent (HSA) synergy scores to quantify performance. In materials science, ChatBattery [58] characterizes physical/structural performance with power conversion efficiency, film quality, and stability. Perovskite-R1 [57] assess the efficacy of the selected additives by fabricating complete solar cell devices and evaluating metrics such as Power Conversion Efficiency (PCE), film quality, and long-term device performance. In social science, Tang et al [66] convert natural language hypotheses into interpretable embeddings used as explanatory variables in a linear regression model to predict societal outcomes. Coefficients are tested for significance, non-significant hypotheses are pruned, and the feedback guides subsequent LLM proposals.

##### T2-S2: Human feedback guided refinement.

Expert review adds domain-specific judgment that complements automated evaluations. In ChatBattery [58], human researchers revise hypotheses after examining experimental outcomes, aligning hypotheses with objectives and constraints when results indicate limited validity or feasibility. CODESCIENTIST [19] generates concise reports indicating whether each hypothesis was confirmed, rejected, or inconclusive, and final expert screening substantially narrows the candidate set.

## 4 End-to-End Workflow

The previous sections examined how individual studies address specific stages and substages of the EXHYTE cycle, providing a granular understanding of each methodological contribution. However, this stage-by-stage analysis does not fully reveal how these strategies can be integrated into an end-to-end scientific discovery workflow. To illustrate how the surveyed components interact in practice, we present two comprehensive systems: AI-Researcher and AI Co-Scientist. These case studies demonstrate how strategies from the EXHYTE framework combine to enable autonomous or semiautonomous discovery, from exploration and hypothesis generation to testing and refinement.

### 4.1 AI-Researcher: an *in silico* workflow

The goal of the AI-Researcher system is to design a complete research pipeline with minimal human intervention capable of systematically targeting the conceptual frontiers of science, deliberately seeking conceptual gaps, contradictory findings, and emerging patterns across literature and implementations. It begins with a curated collection of approximately 15–20 reference papers, along with optional datasets, that form the foundation of knowledge for the system. These sources mirror the intellectual basis of a target publication, providing the conceptual and methodological context from which new discoveries emerge.

#### Explore.

During the exploration stage, AI-researcher establishes the technical and conceptual groundwork for autonomous algorithm design. This process is jointly managed by the *Knowledge Acquisition Agent* and the *Resource Analyst Agent*, supported by their respective sub-agents. The *Resource Analyst Agent* decomposes the research objective into atomic academic concepts, which are fundamental, indivisible elements requiring targeted investigation. Guided by these concepts, the *Knowledge Acquisition Agent* retrieves relevant papers and code repositories from scientific databases, applying multi-criteria filtering to select high-quality sources (E2-S1/S2). The *Paper Analyst* subagent extracts mathematical formulations from LaTeX files using a retrieval-augmented generation (RAG) approach (E3-S1), while the *Code Analyst* identifies corresponding code implementations, establishing explicit bidirectional mappings between theory and code (E3-S3). The resulting structured report provides the empirical and theoretical foundation for downstream reasoning.

#### Hypothesize.

Building on the knowledge assembled during exploration, the *Idea Generator* identifies conceptual gaps, contradictory findings, and emerging patterns across literature and implementations (H1-S3). It employs a Divergent–Convergent Discovery Framework: the divergent phase produces multiple conceptually distinct research directions, while the convergent phase evaluates them using criteria such as novelty, soundness, and transformative potential (H2-S1/S2). The *Advisor Agent* provides expert-style feedback linking theoretical concepts to implementation plans, embodying the human-in-the-loop evaluation principle (H2-S4).

#### Test.

The testing phase operationalizes selected hypotheses through experimental planning, implementation, and iterative refinement. The *Plan Agent* generates a comprehensive development plan, including dataset selection, training/testing methodology, and evaluation metrics (T1-S1). The *Code Agent* translates this plan into executable implementations, verifying dataset usage and experimental consistency (T1-S2). Results are analyzed by the *Experiment Analysis Agent*, which recommends code modifications and further experimentation (T2-S1). Finally, the *Automated Documentation Agent* revises the manuscript using an academic checklist to ensure clarity, reproducibility, and completeness (T2-S3). Together, these stages demonstrate a continuous feedback loop linking hypothesis generation to empirical validation within a coherent *in silico* research pipeline.

### 4.2 AI Co-Scientist: a wet-lab-integrated workflow

The primary goal of the AI-scientist is to accelerate the process of scientific discovery by uncovering new, original knowledge operating within a “scientist-in-the-loop” collaborative paradigm. AI Co-Scientist implements the EXHYTE framework with physical experimentation by coupling LLM reasoning with wet-lab validation. The system accepts multiple input types from the human scientist, most critically a research goal, which may range from concise statements to long documents containing numerous prior publications.

#### Explore.

Upon receiving the research goal, AI Co-Scientist parses it into a *research plan configuration* specifying desired outputs, constraints, and evaluation criteria (E1-S1). This configuration serves as a blueprint for downstream hypothesis generation. The *Generation Agent* then explores relevant literature via web search and retrieval tools (E2-S1/S2) to identify promising directions for novel findings.

#### Hypothesize.

The *Generation Agent* grounds its reasoning in summarized prior work and produces hypotheses and research plans (H1-S3). A *Reflection Agent* simulates a scientific peer-review process, critically examining each hypothesis for correctness, novelty, and evidential grounding (H2-S2). Quick reviews are followed by comprehensive evaluations that integrate external search and evidence retrieval to assess validity. For complex hypotheses, deep verification decomposes each claim into constituent assumptions, which are independently examined to detect invalid components. The *Ranking Agent* employs an Elo-style tournament framework to compare hypotheses through multi-turn debates, scoring them on novelty, correctness, and testability (H2-S1). The top-ranked hypotheses are iteratively refined by an *Evolution Agent* (T2-S1/S3).

#### Test.

The testing stage connects LLM-generated hypotheses to real-world validation. AI Co-Scientist has been deployed in several biomedical domains, demonstrating end-to-end discovery. Notably, the system generated the hypothesis that *chromosomal phage-inducible chromosomal islands (cf-PICIs) hijack phage tails to expand host range*, which was experimentally validated [77]. This “tail-hijacking” mechanism revealed a previously unknown route for horizontal gene transfer in bacteria, advancing understanding of antimicrobial resistance evolution. This case exemplifies how EXHYTE-integrated reasoning—spanning literature exploration, hypothesis generation, prioritization, and refinement—can produce experimentally verified discoveries.

Together, AI-Researcher and AI Co-Scientist illustrate how EXHYTE strategies interconnect across modalities. In both, *Explore* stages ground the system in literature and data; *Hypothesize* stages generate and prioritize testable ideas; and *Test* stages operationalize and refine them through computation or experimentation. These end-to-end workflows demonstrate the practical synthesis of the modular strategies surveyed earlier, showing how autonomous systems can orchestrate the full scientific discovery process from exploration to empirical validation.

## 5 Tools and Datasets for Building EXHYTE Workflows

The preceding sections surveyed strategies for each EXHYTE stage. Here we complement that view with the practical tools, APIs, and datasets that enable those strategies to operate (Table 1 and 2). We group resources by their role in the cycle and note where they most naturally plug into EXHYTE.

### 5.1 Core tools and APIs

#### Literature & data access (E2).

For scientific publications and metadata, commonly used resources include Semantic Scholar [78], PubMed [79], arXiv [80], OpenAlex [81], NASA ADS [83]. Chemical and materials data are typically sourced from PubChem [82] and the Materials Project [60].

#### Text parsing, extraction & normalization (E3).

A variety of tools are employed for text parsing, extraction and normalization. PDF and document content are commonly processed using PyPDF2 [84], SciPDF [85], pymupdf4llm [86], and S2ORC-doc2json [87], while RecursiveCharacterTextSplitter [88] is used for document chunking. HTML content is parsed with BeautifulSoup [89]. For entity extraction and normalization, tools such as BLINK [100], the NER [101] tool, PL-Marker [102], SciCo [103], and ScispaCy [105] are frequently applied.

#### Vector representations, similarity search & retrieval (E2/E3).

A range of embedding generation methods are used to represent text, structured data, molecular properties, and knowledge graph nodes. Text embeddings are commonly produced using models such as Text-Embedding-3 (OpenAI) [106], BERT [91], SciBERT [92], SentenceBERT [93] (all-MiniLM-L6-v2), BAAI/bge-large-en-v1.5 [94], Google-GenerativeAIEmbeddings [95], and jina-embedding-v3 [96]. Molecular similarity is computed using measures such as the Tanimoto coefficient [99] based on fingerprints and ChemBERTa [97] cosine similarity from last hidden state embeddings. For efficient similarity search and retrieval, vector indices and nearest neighbor search libraries such as FAISS [98] are widely used, supporting fast retrieval of semantically or chemically similar items.

#### Cheminformatics and molecular analysis (H2).

Several specialized tools such as RDKit [107] for molecular feature calculation, OmegaFold [108] for protein folding prediction, and SynergyFinder 3.0 [109] for evaluating drug synergy and additivity from experimental data.

### 5.2 Benchmark datasets and evaluation corpora

#### Drug Discovery, Bioactivity, and Clinical:

Several benchmark datasets are widely used in drug discovery and clinical research. DrugBank[22] and KIBA[23] provide molecular and bioactivity data for drug-target interaction prediction, while DAVIS[110] offers drug and protein binding affinity data. Large-scale compound collections such as ZINC[111] (over 230 million purchasable 3D compounds) and ChEMBL[112] (bioactive compounds with properties) are commonly used for training, activity optimization, and modification tasks. Protein-ligand docking performance is assessed using CrossDocked2020[113], and clinical evaluation often relies on MIMIC-CDM[114], which contains 2,400 patients with four abdominal conditions including patient histories, physical exam notes, imaging reports, and laboratory results.

#### Materials Science and Chemistry:

In materials science, datasets such as MP-20 (a subset of Materials Project[60] with 45,231 inorganic crystalline materials), NOMAD[59] (2.9 million materials), Matbench[116], and JARVIS-DFT[115] (61,541 crystal structures with bandgap values) are commonly used for property prediction and generative modeling. OC20-S2EF 2M[117] provides two million catalyst structures for training models such as CatGPT. Additional chemistry-focused benchmarks include TOMATO-Chem[2], for hypothesis rediscovery and ranking from annotated papers, and Superconductor Optimization (SPO)[17], which contains 26,321 superconducting materials with chemical formulas and critical temperatures.

#### Auxiliary Multi-domain Testbeds:

Various multi-domain datasets are used for evaluation in NLP, AI reasoning, and behavioral tasks. BUSINESS SHOES[118] evaluates binary classification for shoe suitability, and DECEPTIVE REVIEWS[119] contains genuine and fake hotel reviews for deception detection. HEADLINE POPULARITY[120] and TWEET POPULARITY[121] assess popularity prediction of headlines and tweets, respectively, while DREADDIT[122] and PERSUASIVE PAIRS[123] focus on mental stress detection and persuasive argument prediction. Systematic review and reasoning datasets include RCT Summaries[124], which links randomized controlled trials to PubMed papers, and HypoGen[42], containing approximately 5,500 structured problem-hypothesis pairs from NeurIPS 2023 and ICLR 2024 papers, used for fine-tuning and evaluation.

### 5.3 How these resources connect to EXHYTE

**E2 (Data Retrieval)** is powered by literature and dataset retrieval APIs(Semantic Scholar[78], PubMed[79], arXiv[80], OpenAlex[81], PubChem[82]) and efficient similarity search tools such as FIASS[125], Tanimoto coefficient[99] etc. **E3 (Knowledge Assembly)** leverages PyPDF2[84], SciPDF[85] for parsing documents, RecursiveCharacterTextSplitter[88] for document chunking, pymupdf4llm[86], S2ORC-doc2json[87] for documents formatting, BLINK[100], NER[101], PL-Marker[102], SciCo[103] for entity extraction and normalization. **H2 (Prioritization)** draws on similarity metrics (Tanimoto[99], ChemBERTa[97]) and benchmark corpora (e.g., TOMATO-Chem[2], Matbench[116]) to score plausibility and novelty against known neighborhoods. **T1–T2 (Test & Refinement)** use domain datasets (OC20-S2EF[117], JARVIS-DFT[115], MIMIC-CDM[114], ZINC[111]) to instantiate executable protocols, measure performance with domain metrics, and iterate using empirical feedback.

A typical *in silico* screening loop begins by validating textual inputs (molecular SMILE string using RDKit[107]) and PyPDF2[84], SciPDF[85], and pymupdf4llm[86] are used for related PDF documents parsing and content extraction supported by structured conversion pipelines such as S2ORC-doc2json[87] and the RecursiveCharacterTextSplitter[88]. Candidate representations are then retrieved through embedding-based similarity search, using FAISS[98] for vector indexing and retrieval, Tanimoto[99] fingerprint similarity or ChemBERTa[97] latent-space cosine similarity for molecular comparison, and text embeddings generated via models such as Text-Embedding-3[95], SciBERT[92], or BAAI/bge-large-en-v1.5[94]. Task-relevant features are extracted and normalized via entity recognition and linking tools including BLINK[100], NER[101], PL-Marker[102], SciCo[103], and ScispaCy[105]. Candidate hypotheses or molecular designs are subsequently evaluated on domain-specific benchmark datasets—such as Matbench[116] or MP-20[60] providing quantitative feedback for **H2** prioritization and informing iterative refinement under **T1–T2**. This loop enables EXHYTE systems to continuously integrate retrieval (E2), structured knowledge assembly (E3), and hypothesis ranking (H2) within a unified testing and improvement cycle.

Together, these tools and datasets provide the material on which the EXHYTE strategies operate, enabling systems to move from retrieval and assembly to principled prioritization, testing, and refinement across chemistry, materials, biomedicine, and general scientific reasoning.

## 6 Conclusion and Future Outlook

This survey reframes AI for scientific discovery using the EXHYTE cycle, a *process-centric* framework positioning LLMs and agents within *Explore*, *Hypothesize*, and *Test* stages. Analyzing recent literature through this lens unifies diverse research, identifies key advances and gaps, and reveals opportunities for integrating emerging approaches into complete discovery workflows.

Our synthesis finds that the EXPLORE stage is the most mature, driven by strong advances in literature retrieval, RAG pipelines, knowledge-graph construction, and vector-based representations. However, this maturity is uneven: nearly all systems explore *literature* as the primary source of evidence, whereas only a small fraction examine *real scientific data* to surface patterns, anomalies, or knowledge gaps. This imbalance limits the ability of current systems to initiate genuinely data-driven discovery. In contrast, the HYPOTHESIZE stage remains underdeveloped; although many systems can generate hypotheses, few provide rigorous mechanisms for evaluating, prioritizing, or iteratively refining them based on empirical outcomes. The TEST stage, particularly experimental design and closed-loop validation, likewise lags behind, with most efforts remaining in simulation or other *in silico* settings. Together, these gaps underscore the need for deeper integration across data analysis, symbolic reasoning, statistical modeling, and physical experimentation to advance toward truly autonomous discovery workflows.

The EXHYTE mapping highlights a methodological shift from domain-specific pipelines toward cross-domain, transferable discovery frameworks. As agents gain multimodal reasoning and memory, the boundaries between retrieval, reasoning, and experimentation will blur, enabling systems that learn across scientific contexts. Concurrently, the human-AI interface remains central. The most effective systems model the epistemic structure of inquiry, distinguishing evidence from interpretation and maintaining transparency for human oversight.

Looking ahead, the EXHYTE framework serves as both a roadmap and a research agenda. Technical progress requires linking multimodal reasoning, simulation, and autonomous experimentation into adaptive, self-correcting workflows. Conceptually, new metrics are needed beyond accuracy or novelty to measure iterative coherence, hypothesis refinement, and explanatory depth. The future of AI-driven discovery lies not in replacing scientists, but in creating reasoning collaborators. These systems will explore knowledge, test hypotheses, and refine understanding in a continuous loop, marking a transition within the EXHYTE framework from task automation to integrative scientific intelligence.

## Supplementary Files

This is a list of supplementary files associated with this preprint. Click to download.

- supplementaryfile.xlsx

The online version contains the supplementary file that summarizes all 83 papers, indicating with check marks which strategies were employed by each paper. The file is also available at https://webapps.crc.pitt.edu/exhyte/.

## Figures and Tables

**Table 1:** AI tools and APIs supporting the EXHYTE workflow

Category	Tools/APIs
**Literature & Data Retrieval APIs (E2)**	Semantic Scholar [78], PubMed [79], arXiv [80], OpenAlex [81], PubChem [82], NASA ADS [83], Materials Project [60]
**Text Parsing & Normalization (E3)**	PyPDF2 [84], SciPDF [85], pymupdf4llm [86], S2ORC doc2json [87], RecursiveCharacterTextSplitter [88], BeautifulSoup [89]
**Embedding & Representation (E2/E3)**	OpenAI text embeddings [90], BERT [91], SciBERT [92], SentenceBERT [93], BAAI/bge-large-en-v1.5 [94], Google-GenerativeAIEmbeddings [95], jina-embedding-v3 [96], ChemBERTa [97]
**Similarity Search & Indexing (E2/H2)**	FAISS [98], Tanimoto [99]
**Text Mining & Knowledge Graph Construction (E3)**	BLINK [100], NER (Entity Linker) [101],PL-Marker [102], SciCo [103], SciSpacy [104]

**Table 2:** Benchmark datasets across domains

Category	Subcategory	Dataset Names
**Drug Discovery & Bioactivity**	Drug-target interaction	**DrugBank** [22], **KIBA** [23], **DAVIS** [110]
	Large compound collections	**ZINC** [111], **ChEMBL** [112]
	Protein-ligand docking	**CrossDocked2020** [113]
	Clinical / patient data	**MIMIC-CDM** [114]
**Materials Science & Chemistry**	Inorganic crystal structures	**MP-20** [60], **NOMAD** [59], **JARVIS-DFT** [115]
	Materials property prediction	**Matbench** [116]
	Catalyst structures	**OC20-S2EF 2M** [117]
	Chemistry hypothesis	**TOMATO-Chem** [2]
	Superconducting materials	**Superconductor Optimization (SPO)** [17]
**Multi-domain/NLP/AI Reasoning**	Binary classification / suitability	**BUSINESS SHOES**[118]
	Deception detection	**DECEPTIVE REVIEWS** [119]
	Popularity prediction	**HEADLINE POPULARITY** [120], **TWEET POPULARITY** [121]
	Mental stress & persuasion	**DREADDIT** [122], **PERSUASIVE PAIRS** [123]
	Systematic review & literature reasoning	**RCT Summaries** [124], **HypoGen** [42]
